# High-Fidelity Visual Long-Term Memory within an Unattended Blink of an Eye

**DOI:** 10.3389/fpsyg.2017.01859

**Published:** 2017-10-20

**Authors:** Christof Kuhbandner, Elizabeth A. Rosas-Corona, Philipp Spachtholz

**Affiliations:** ^1^Department of Psychology, University of Regensburg, Regensburg, Germany; ^2^Department of Psychology, University of Munich, Munich, Germany

**Keywords:** visual long-term memory, perceptual memory, capacity, attention, inattentional blindness

## Abstract

What is stored in long-term memory from current sensations is a question that has attracted considerable interest. Over time, several prominent theories have consistently proposed that only attended sensory information leaves a durable memory trace whereas unattended information is not stored beyond the current moment, an assumption that seems to be supported by abundant empirical evidence. Here we show, by using a more sensitive memory test than in previous studies, that this is actually not true. Observers viewed a rapid stream of real-world object pictures overlapped by words (presentation duration per stimulus: 500 ms, interstimulus interval: 200 ms), with the instruction to attend to the words and detect word repetitions, without knowing that their memory would be tested later. In a surprise two-alternative forced-choice recognition test, memory for the unattended object pictures was tested. Memory performance was substantially above chance, even when detailed feature knowledge was necessary for correct recognition, even when tested 24 h later, and even although participants reported that they do not have any memories. These findings suggests that humans have the ability to store at high speed detailed copies of current visual stimulations in long-term memory independently of current intentions and the current attentional focus.

## Introduction

Imagine that you are walking along a street together with a friend, focusing your attention completely on your conversation. While walking and talking, your eyes wander around, randomly fixating for less than a second on visual objects passing by, without you noticing the information that reaches your eyes at all. Twenty-four hours later, someone asks you unexpectedly about your memories for the objects your eyes had briefly fixated on during that walk. How many objects would you remember? And if you remembered any object at all, how detailed would the memory be? The aim of the present study was to examine these questions.

The question of what is stored in long-term memory from current sensations has intrigued humans for centuries (e.g., Burnham, 1888; Loftus and Loftus, 1980). In view of the apparent problem that an overwhelming amount of information would have to be stored and handled if all of the information reaching our senses at any moment persisted in memory, common wisdom over time has been that only a small part of the information leaves a durable memory trace whereas the rest is quickly lost, an assumption that is reflected in prominent theories which have been developed over time (e.g., Atkinson and Shiffrin, 1968; Wolfe, 1999; Cowan, 2001; Lavie, 2010). More specifically, the common assumption is that primary sensations from the environment are only temporarily stored in large-capacity sensory-memory systems. As processing at later stages is strongly limited in capacity, only a small part of the information stored in sensory memory can be read out for further processing and final storage in long-term memory. The mechanism underlying selection for further processing is assumed to be attention. Only information to which attention is directed is further processed whereas unattended information is no longer available after short amounts of time, an assumption that lies also at the heart of classic early-selection theories (Broadbent, 1958; Treisman, 1969).

In fact, such a view seems to be supported by abundant empirical evidence. The existence of sensory memory and the seemingly rapid decay of unattended sensory information were first demonstrated by Sperling (1960). He showed that when observers were asked to report as many stimuli as possibly from a briefly presented stimulus array, they were able to report only about four stimuli, a limit which is believed to reflect the limited capacity of visual working memory (e.g., Luck and Vogel, 1997; Cowan, 2001). Intriguingly, if a cue was presented after the offset of the array that directed attention to four stimuli of the array that should be reported, observers were able to report the cued stimuli almost perfectly. As the to-be-reported stimuli were not known until the cue was presented, this finding implies that at the time of the cue many more stimuli are available than are subsequently stored in visual working memory. However, with increased delay of the cue, performance quickly decreased, and the cue was no longer helpful after less than a second, which suggests that unattended sensory information decays within less than a second (for a review for the numerous replications, see Cowan, 2008). In more recent research, the very rapid decay of sensory memory has been questioned by studies where memory was measured by the ability to detect a change in the presented stimulus array (i.e., change detection paradigm) rather than by the ability to recollect stimuli from memory, showing that cues can be helpful for up to 4 s (the so-called retro-cue effect; e.g., Lepsien et al., 2005; Sligte et al., 2008). However, beyond demonstrating that the life-time of sensory memory may be slightly longer, these studies still suggest that information stored in sensory memory is fragile and only protected from decay when attention is directed to it (e.g., Sligte et al., 2008).

The assumption that unattended information is not stored beyond the current moment seems to also be supported by numerous recent studies in the domain of long-term memory. In these studies, to rule out the methodological problem that the processing of the task-relevant stimuli may leave some unused capacity that then may unintentionally spill over to task-irrelevant stimuli, high attentional load tasks were used. Observers saw a rapid stream of pictures overlapped by words, and were instructed to attend to either the pictures or the words and detect stimulus repetitions in the attended domain. In all of the existing studies using such high attentional load tasks, it was consistently found that observes showed a null memory effect for the unattended stimuli in a subsequent recognition test (Rees et al., 1999; Ruz et al., 2005a,b; Butler and Klein, 2009; Lavie et al., 2009; Hoffman et al., 2011). Thus, taken together, it appears to be common wisdom that only attended information is stored in long-term memory whereas unattended information decays rapidly and is no longer reportable after short periods of time.

However, using the above high attentional load paradigm, over time, a few findings have been reported that make it appear possible that this common wisdom may actually not be true. First, while replicating the finding that no explicit awareness of unattended information is apparent in recognition tests, Butler and Klein (2009) reported increased perceptual priming effects for unattended stimuli in an implicit perceptual memory test, indicating that unattended information may be retained at least in the form of perceptual representations. Indeed, this finding is reminiscent of findings in the domain of repetition priming, demonstrating that a brief exposure to a visual image without mentioning that memory will be tested later leads to a processing benefit when the image is encountered again after several days, months, or even years (Mitchell and Brown, 1988; Mitchell, 2006). Second, while again replicating the finding that no explicit awareness of unattended information is apparent in recognition tests, Hoffman et al. (2011) reported that participants were less confident in their “no” responses to unattended old stimuli compared to the “no” responses to new stimuli. Such a finding provides preliminary evidence that the null effects found in previous studies may actually not reflect absent explicit memory for unattended stimuli, but instead the fact that memory tests have been used that were insufficiently sensitive to detect it.

**FIGURE 1:**
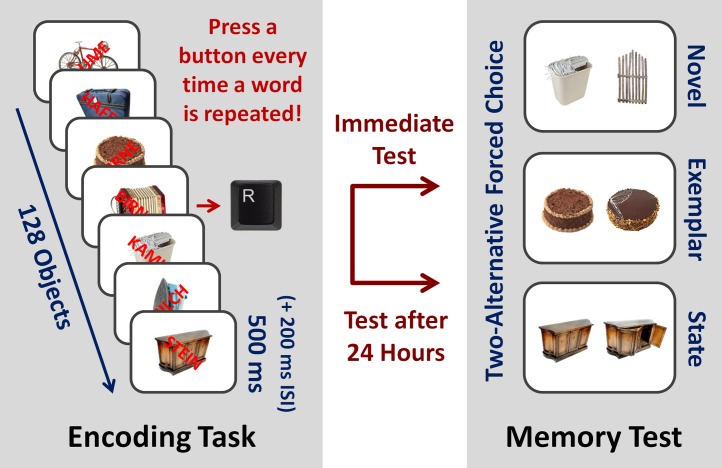
Memory Paradigm. Participants were shown a rapid stream of 128 different real-world object pictures overlapped by different words that were randomly rotated from trial to trial clockwise or counterclockwise with the instruction to press a button every time a word was repeated (presentation time of stimuli: 500 ms, interstimulus interval: 200 ms, two cycles). No mention was made that memory for the object pictures would be tested later. Memory for half of the object pictures was tested in a surprise recognition test immediately afterward, the other half was tested in a second recognition test after 24 h. A two-alternative forced choice test was used with foil objects varying in similarity (novel vs. exemplar vs. state).

The aim of the present study was to examine whether actually much more from current sensations is stored in long-term memory than currently believed by using a more sensitive recognition test than in previous research. Following the procedure used in previous studies, observers were shown a rapid stream of pictures of real-world objects overlapped by words, with the instruction to attend to the words and to press a button every time a word was repeated (for an illustration, see **Figure 1**). To preclude any strategic encoding processes, it was not mentioned that memory for the objects would be tested later. However, unlike in previous studies where recognition was measured by single item old/new recognition tests, we used a two-alternative forced choice recognition test where a previously seen object was paired with a foil object that had not been presented before, with the instruction to select the previously seen object. As shown in previous research, compared to old/new recognition tests that heavily rely on recollective experience, such a memory test more sensitively measures the actual amount of information stored in memory (Holdstock et al., 2002; Cunningham et al., 2015).

Beyond examining the pure existence of long-term memory representations for unattended stimuli, we addressed three further questions. First, to additionally measure the fidelity of stored representations, we varied the similarity between the previously presented objects and the foils in three steps (novel vs. exemplar vs. state; for an illustration, see **Figure 1**; for details see the method section), following previous research in the domain of visual long-term memory (Brady et al., 2008). Second, to additionally measure the durability of stored representations, half of the objects were tested immediately after the perception task, and the other half were tested after 24 h. Third, to additionally examine whether participants were aware of their memory abilities, for each response in the memory test, participants were asked to indicate whether a response was made based on recollective experience or whether they guessed. In view of recent findings that participants can show relatively substantial levels of recognition memory in two-alternative forced choice recognition tests despite believing that they are simply guessing (Voss et al., 2008; Craik et al., 2015), it may be that the involved memory system operates below awareness.

## Materials and Methods

### Participants

Forty-one undergraduate students (35 females; mean age = 20.1 years, *SD* = 3.1) participated for course credit. The sample size was based on a power analysis (G^∗^Power 3.1.718; Faul et al., 2007) to have sufficient power (0.80, alpha = 0.05, one-tailed) in order to detect small-to-medium sized effects (*d* = 0.4). All participants provided written informed consent and reported normal or corrected-to-normal vision acuity and normal color vision. Data from one participant were excluded due to poor word-repetition detection performance (below 75%). Including this participant did not change any of the reported results. All data exclusions, all manipulations, and all measures in the study are reported.

### Materials, Design, and Procedure

The procedure of the study followed the paradigm introduced by Rees et al. (1999) and involved an incidental encoding task, an immediate recognition test that was conducted directly after the encoding task, and a delayed recognition test that was conducted after 24 h. In the incidental encoding task, a rapid stream of 128 sequentially presented stimuli was presented two times with a short break of 1 min in between. All Stimuli were presented on a white background at the center of a screen for 500 ms with an interstimulus interval of 200 ms using E-Prime 2.0 (PST, Pittsburgh, PA, United States). Each stimulus consisted of an object picture (approximately 5° of visual angle) superimposed with a five-letter German noun (uppercase letters and colored in red) that was unrelated to the objects. The stream started and ended with four filler stimuli that were not included in the later memory tests to control for primacy and recency effects. In between, 120 target object pictures were presented in random order. The 120 object pictures were taken from the study by Brady et al. (2008). To manipulate word repetitions, two pseudorandom word orderings were generated where word repetitions occurred on average once every five items (24 repetitions per presentation sequence). The words were randomly rotated from trial to trial by 45° clockwise or counterclockwise, and they were always shown in different orientations on repetition trials. Participants were instructed to attend to the words, and to press a button every time a word was repeated. To acquaint participants with the task, they initially practiced the repetition detection of words on a stream of 20 stimuli that were not used in the later experiment.

In both the immediate and the delayed recognition memory tests, two objects were presented on the screen, one previously seen old object picture, and one new foil object picture. To vary the similarity between previously presented objects and foils, from the 120 previously presented object pictures, 40 were paired with pictures from an object of a different category (novel), 40 were paired with a picture from a physically similar object from the same basic-level category (exemplar), and the remaining 40 were paired with a picture from the same object that differed in the shown state or pose (for details see Brady et al., 2008). Observers were instructed to indicate which of the objects they had seen before in a two-alternative forced-choice task. Participants were allowed to proceed at their own pace. They were instructed to guess if they could not base their response on recollective experience, and after each response, they were asked to indicate whether they had guessed. In the immediate recognition test, half of the 120 target object pictures (20 novel, 20 exemplar, 20 state) were tested. In the recognition test after 24 h, all 120 target object pictures were tested. The assignment of object pictures to the immediate test was counterbalanced across participants. In half of the trials, the foil object was shown on the left, in the other half on the right; the order of trials was randomized. After the immediate test, participants were asked to report whether they had expected that their memory for the objects would be tested later.

### Statistical Analysis

As a correct response in a two-alternative forced choice test can reflect not only a correct response but also a fortunate guess, we corrected the observed percentage correct (PC_Observed_) for each participant for the effects of random guessing, using the formula PC_Adjusted_ = 2 ^∗^ PC_Observed_ - 100. The adjusted memory performance (PC_Adjusted_) estimates how often observers truly remember an object, after accounting for fortunate guesses (see Brady et al., 2013). To determine whether memory performance was above chance, one-sample *t*-tests with one-tailed alpha level were used for data analysis. In the recognition test after 24 h, data were separately analyzed for objects that had not been tested in the immediate recognition test, and objects that had already been tested.

## Results

### Incidental Encoding Task

Mean word repetition detection performance in the incidental encoding task was high (*M* = 0.95, 95% CI [93.2, 96.0]), indicating that all participants fully focused their attention on the words. The post-experiment questionnaire confirmed that none of the participants expected that their memory for the stimuli would be tested later.

### Recognition Memory

**FIGURE 2:**
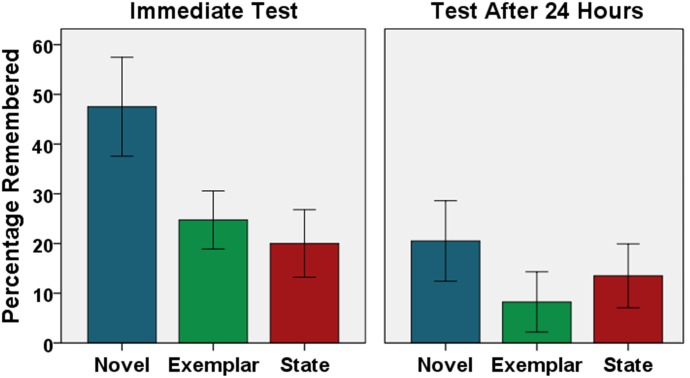
Recognition Memory Performance. The percentage of correctly remembered object pictures (corrected for guessing) is shown as a function of delay of the test (immediate vs. 24 h) and type of foil object (novel vs. exemplar vs. state). Error bars represent 95% confidence intervals.

The results for the immediate and delayed recognition memory tests are shown in **Figure 2**. The height of the bars shows the percentage of objects that was correctly remembered, after accounting for fortunate guesses. In the immediate test, observers showed strong object memory, even when subtle details had to be remembered (Novel: *M* = 47.5%, 95% CI [37.5, 57.5], *t*(39) = 9.55, *p* < 0.001, *d* = 1.53; Exemplar: *M* = 24.8%, 95% CI [18.9, 30.6], *t*(39) = 8.40, *p* < 0.001, *d* = 1.36; State: *M* = 20.0%, 95% CI [13.2, 26.8], *t*(39) = 5.81, *p* < 0.001, *d* = 0.94). In the delayed recognition test, even when memory for the objects was the first time tested after a delay of 24 h, still substantial memory was found (Novel: *M* = 20.5%, 95% CI [12.4, 28.6], *t*(39) = 5.00, *p* < 0.001, *d* = 0.81; Exemplar: *M* = 8.3%, 95% CI [2.2, 14.3], *t*(39) = 2.59, *p* = 0.007, *d* = 0.44; State: *M* = 13.5%, 95% CI [7.1, 19.9], *t*(39) = 4.10, *p* < 0.001, *d* = 0.67). Memory performance for objects that had already been tested in the immediate recognition test was high as well (Novel: *M* = 36.8%, 95% CI [27.5, 46.1], *t*(39) = 8.03, *p* < 0.001, *d* = 1.27; Exemplar: *M* = 22.0%, 95% CI [14.0, 30.0], *t*(39) = 5.54, *p* < 0.001, *d* = 0.88; State: *M* = 10.3%, 95% CI [3.1, 17.4], *t*(39) = 2.91, *p* = 0.003, *d* = 0.46).

Regarding participants’ awareness of their memory abilities, for most of their responses, participants reported that they simply had guessed (immediate test: *M* = 76.9% of trials, 95% CI [72.5, 81.3]; test after 24 h for objects that had not been tested in the immediate test: *M* = 94.5% of trials, 95% CI [92.3, 96.8]). Analyzing results only for guess trials revealed still substantial object memory, both in the immediate test (Novel: *M* = 26.3%, 95% CI [13.1, 39.5], *t*(39) = 3.95, *p* < 0.001, *d* = 0.64; Exemplar: *M* = 10.7%, 95% CI [3.5, 17.9], *t*(39) = 2.87, *p* = 0.003, *d* = 0.48; State: *M* = 14.2%, 95% CI [7.7, 20.8], *t*(39) = 4.23, *p* < 0.001, *d* = 0.69) and in the test after 24 h (Novel: *M* = 16.5%, 95% CI [8.5, 24.6], *t*(39) = 4.02, *p* < 0.001, *d* = 0.66; Exemplar: *M* = 7.0%, 95% CI [1.0, 12.9], *t*(39) = 2.18, *p* = 0.018, *d* = 0.37; State: *M* = 13.9%, 95% CI [7.2, 20.6], *t*(39) = 4.03, *p* < 0.001, *d* = 0.66).

## Discussion

Several prominent theories that have been developed over time have proposed that only attended information leaves a durable memory trace whereas unattended information is not stored beyond the current moment (e.g., Broadbent, 1958; Atkinson and Shiffrin, 1968; Wolfe, 1999; Cowan, 2001; Lavie, 2010). Some of the most convincing results seem to come from studies where observers were shown a rapid stream of words superimposed on real-world object pictures with the instruction to attend to either the pictures or the words and detect stimulus repetitions in the attended domain, without mentioning that memory would be tested later. Because such a task poses high demands on processing capacity and because participants did not expect a subsequent memory test, an unintentional spill over of attention to task-irrelevant stimuli is minimized. All existing studies have consistently found that observes showed a null memory effect for the unattended stimuli in a subsequent memory test (Rees et al., 1999; Ruz et al., 2005a,b; Butler and Klein, 2009; Hoffman et al., 2011).

However, in these previous studies, single item old/new recognition tests have been used. By using instead a more sensitive two-alternative forced choice recognition test, the present findings challenge the view that unattended stimuli are not stored beyond the current moment. When a previously presented picture was paired with a new picture from an object of a different category (novel condition), almost half of the presented unattended pictures were correctly recognized (recognition rates corrected for guessing). Even more intriguingly, even when detailed feature knowledge was necessary for correct recognition (exemplar and state conditions), recognition performance was substantially above chance. And most intriguingly, even when participants’ memory for the unattended pictures was tested the first time 24 h later, recognition performance was still substantially above chance. These findings demonstrate that humans unintentionally store at high speed detailed copies of current visual stimulations in long-term memory for at least 24 h independently of the current focus of attention.

Interestingly, recognition performance was high even although participants reported that they do not have any memories and that they simply guessed. Such a finding supports recent findings demonstrating the phenomenon of recognition without awareness (Voss et al., 2008; Craik et al., 2015), and may provide an explanation why the existence of long-term memory representations for unattended stimuli has been hitherto overlooked. Indeed, the present findings remind of the phenomenon of “blindsight” in which cortically blind individuals are able to identify visual stimuli when they are prompted to guess (Kentridge et al., 1999). Interestingly, these persons do not show any kind of confidence in their abilities even when informed about it, suggesting that they are both unaware and not convincible about their abilities. Thus, similar to blindsight individuals, it may be almost impossible to discover by everyday introspection that there is actually much more stored in visual long-term memory than consciously experienced.

One interesting question concerns the nature of the visual long-term memory representations that were formed for the unattended pictures of real-world objects. Basically, from a perceptual perspective, two qualitatively different processing steps are involved when visual objects are initially represented in the cognitive system, and both of their outputs may provide the basis on which objects are stored. First, signals from the retina are analyzed to extract visual features such as orientation, colors, and so forth, a process by which highly detailed representations of independent features are created that are closely linked to the physical properties of the visual objects. Second, the independent feature representations are integrated into coherent object representations, leading to the phenomenal experience of a visual scene that is segregated into coherent objects (e.g., Riesenhuber and Poggio, 1999; Serences and Yantis, 2006). As it is commonly assumed that attention is required for the binding of features into coherent object representations (e.g., Treisman and Gelade, 1980), it seems that the unattended real-world objects in the present study were stored in the form of independent features representations. Indeed, previous research has shown that low-level feature information such as spatial frequency information can be retained with high precision in long-term memory (Magnussen and Dyrnes, 1994; Magnussen et al., 2003), and that the quality of object representations in visual long-term memory can be predicted from early preattentive brain activities (Spachtholz and Kuhbandner, 2017). Thus, the present findings support early speculations that there might be a sensory long-term memory system that stores information about currently processed visual information (Johnson, 1983).

Another question concerns the possible limits of the ability to store detailed copies of current visual stimulations in long-term memory. On the one hand, as the objects in the present study were visually overlapped by words to withdraw the observers’ attention from the objects, it seems likely that the observed memory performance even underestimates what is stored from objects when they are not partly hidden by artificial irrelevant stimuli. On the other hand, previous research provides some hints that there may be both spatial and temporal limits. Regarding spatial limits, as the unattended stimuli in the present study were presented foveally, it may be that unattended peripheral stimuli are not stored in long-term memory. Indeed, preliminary evidence comes from a study by Lavie et al. (2009) where no object memory in a two-alternative forced choice test was observed when to-be-attended stimuli surrounded to-be-ignored object pictures. Regarding temporal limits, previous research has shown that the pictures in a rapid serial visual presentation task have to be presented long enough to assure that they can be recognized later. Although observers are able to detect a picture on the basis of meaning possibly even at presentation durations as brief as 13 ms (Potter et al., 2014; but see Maguire and Howe, 2016), subsequent recognition memory seems to be poor if the presentation rate is below 500 ms (Potter and Levy, 1969; for a review see Potter, 2012). Thus, it may be that unattended stimuli are not stored if presented at presentation rates below 500 ms. However, since mainly single item old/new recognition tests have been used in the studies on recognition memory for rapidly presented pictures, potential temporal limits have to be explored in future research.

To summarize, the present findings may provide a starting point to rethink current models of memory storage as it seems to be the case that much more information is stored in long-term memory than previously believed. Furthermore, the present findings may have important implications for applied settings such as eyewitness testimony because visual memories about past events may exhibit a much higher degree of details when appropriately tested. Thus, the present study may open new interesting avenues for both basic and applied future research.

## Ethics Statement

This study was carried out in accordance with the ethical standards at the University of Regensburg where the experiments were conducted with written informed consent from all subjects. All subjects gave written informed consent in accordance with the Declaration of Helsinki.

## Author Contributions

CK developed the research idea and designed the study. ER-C and PS programmed the computer task. ER-C collected data. CK, ER-C, and PS analyzed the data and interpreted the results. CK drafted the manuscript, and PS provided critical revisions. All authors approved the final version of the manuscript for submission.

## Conflict of Interest Statement

The authors declare that the research was conducted in the absence of any commercial or financial relationships that could be construed as a potential conflict of interest.
